# Evaluation of a Community-Based Student-Led Health Equity Curriculum

**DOI:** 10.7759/cureus.103908

**Published:** 2026-02-19

**Authors:** Manish Kumar, Eirene Fithian, Carlyn Wisherop, Martin Shapiro

**Affiliations:** 1 Department of Medicine, Weill Cornell Medicine, New York, USA

**Keywords:** community-based medicine, curriculum, medical education assessment, preventative care strategies, student-run organizations

## Abstract

Background

The Community Perspectives in Medicine (CPIM) elective at Weill Cornell Medical College was developed to address curricular gaps in teaching social determinants of health (SDOH). The course connects first-year students with community-based organizations and health justice topics through interactive sessions. In 2023, CPIM was redesigned to emphasize nationally debated health justice issues and to include a community engagement project. This study evaluated the redesigned course’s impact on students’ knowledge, confidence, and advocacy skills.

Methodology

Students attended seven weekly one-hour lectures led by community health advocates, each followed by a 30-minute student-led discussion. Students completed a community engagement project with a local organization and presented their experiences in the final session. Pre- and post-course surveys assessed confidence in course topics and intent to incorporate advocacy into future practice using a five-point Likert scale. Differences were analyzed with Wilcoxon rank-sum tests. Qualitative feedback captured perceived impact and key learning themes.

Results

In 2023, confidence ratings increased significantly from 2.71 to 4.69 on a five-point scale (p < 0.001), with the greatest gains in understanding single-payer healthcare. In 2024, ratings increased from 2.62 to 3.72 (p < 0.001), with the largest improvement in understanding political advocacy in medicine. Qualitative responses highlighted enhanced motivation to pursue advocacy and stronger connections to marginalized communities.

Conclusions

CPIM effectively increases students’ confidence in engaging with SDOH and advocacy. Its success highlights its potential as a model for integrating community-centered, justice-focused education into medical training.

## Introduction

Medical education increasingly recognizes the importance of preparing students to address both individual patient needs and the broader social and community factors that influence health outcomes [1]. Despite increasing efforts to include social determinants of health (SDOH) in formal medical school curricula [2,3], programs still do not prioritize teaching SDOH to the same level as clinical sciences [4]. Studies suggest that many faculty members feel unprepared to teach SDOH due to limited training and institutional support, exacerbating the state of health equity education [5]. This environment highlights the need for student-led medical education on health equity.

Medical students developed Community Perspectives in Medicine (CPIM) at Weill Cornell Medical College in 2014 to meet this need, exposing students to real-world issues surrounding SDOH, health disparities, and the role of advocacy in healthcare [6]. By incorporating guest lectures and partnerships with community-based organizations (CBOs), CPIM enriched traditional medical training through direct engagement with structural factors that shape health.

The original implementation of CPIM demonstrated the effectiveness of integrating community engagement and advocacy into the medical curriculum. Initial evaluations of the course found that students gained significant awareness of how socioeconomic, cultural, and environmental factors shape patient health. By hearing from CBO representatives, students learned about healthcare disparities from those experiencing them firsthand. Initial evaluations also identified opportunities to strengthen the course, specifically regarding students’ confidence in acting on these insights and maintaining an advocacy-oriented perspective throughout their training [6].

In response, we restructured CPIM in 2023 to include topics highlighting health justice issues that have been the focus of national discussion and debate and required students to partner with New York City advocacy organizations. These changes reflect growing recognition that healthcare professionals must be equipped to understand and address the structural factors driving health disparities. Physicians, by virtue of their trusted role and firsthand perspective on health inequities, are well-positioned to engage in these conversations and to lead efforts toward more equitable systems. In emphasizing civic health and the role of civic engagement in shaping community well-being, this approach highlights community engagement as a critical element of medical education [7,8].

This study expands on CPIM’s initial evaluation and responds to calls for outcomes-based research in medical education [9]. We also share updated data on how students perceive community health and their role in advocacy before and after participation in the course, providing insights into the effectiveness of community-centered curricula that reflect the evolving landscape of healthcare activism. Lastly, we aim to provide detailed information on the development of our course to empower student-led initiatives to improve the health equity curricula at medical schools nationwide.

## Materials and methods

Study design

We conducted an evaluation comparing pre- and post-survey results of the CPIM elective offered during the Fall 2023 and Fall 2024 semesters. Our primary quantitative outcome was a change in students’ self-reported confidence in knowledge of course topics, which was collected using a pre-course and post-course survey. Surveys were administered anonymously and were not linked at the individual level. Secondary quantitative outcomes included students’ self-reported willingness and readiness to integrate health advocacy into their clinical practice. Qualitative outcomes included students’ perception of the course’s impact on their understanding of health justice, motivation to engage in advocacy, and the influence of their community engagement projects on their professional development.

Course administration

We administered CPIM as a seven-week, non-credit-granting elective for first-year medical students at Weill Cornell Medical College during the fall semester. We conducted the course in person, with weekly 90-minute sessions. We allocated the first 45-60 minutes to lectures, followed by small group discussions in the remaining time. We facilitated the course with a 2-3-person leadership team, comprising a “Course Director” and 1-2 “Logistics Coordinators,” all of whom were medical students. Course Directors were responsible for coordinating with speakers, emailing students and speakers, and facilitating sessions, while Logistics Coordinators requested a budget, finalized room reservations, and coordinated food for sessions. The entire leadership team was involved in identifying speakers and course evaluation. The course also had a faculty advisor who provided guidance on curriculum development and supported student leadership throughout the semester.

Every week, the CPIM leadership team invited speakers engaged in community-based activism to provide lectures on specific health justice issues and their related activism. Topic selection was guided by the Centers for Disease Control and Prevention (CDC) framework for SDOH, which identifies key domains such as healthcare access, education, economic stability, social and community context, and neighborhood environment. Within these domains, the leadership team prioritized emerging health justice issues that were the focus of national debate or reflected unmet educational needs within our curriculum. One session each year is dedicated to discussing students’ community engagement projects and how they can integrate advocacy into their future careers.

We funded the course using a $1,200 grant from the Diversity Center of Excellence at Weill Cornell Medical College. In addition, we received annual funding from the Medical School Executive Council ($500 in 2023 and $700 in 2024). We used course funds to pay speakers an honorarium and provide dinner for students.

Speaker recruitment

In alignment with previous years, we began recruiting speakers for CPIM two to three months before the start of the course, following topic selection. We identified speakers through three major approaches. These approaches include leveraging personal connections with individuals and organizations, engaging faculty involved in health activism, and identifying grassroots CBOs working on pre-selected topics and inviting their participation.

We shared our goals for CPIM with identified speakers (see Appendix A for an email template). Once confirmed, we collected speaker biographies for introductions at the beginning of each session. We provided speakers with the option to lecture in person or over Zoom; however, students were expected to report in person for all sessions. In recognition of their time, we offered speakers a gift card ($50 in 2023, $75 in 2024). Several speakers declined compensation.

Final topics for the Fall 2023 cohort included single-payer healthcare, chronic illness, hospital surveillance, detainee health, indigenous health, and pursuing advocacy as a healthcare professional.

Between Fall 2023 and Fall 2024, the CPIM leadership team decided to alter several of the topics for the course. This decision was made in alignment with the revised course’s objective to highlight health justice issues that have been the focus of national discussion and debate in a particular year.

Final topics for the Fall 2024 cohort included single-payer healthcare, health in humanitarian crises, the role of community health clinics/federally qualified health centers, detainee health, gender-affirming care, and pursuing advocacy as a healthcare professional.

Student recruitment

We recruited first-year medical students via Weill Cornell’s annual student activities fair and through email. About one month before the course, we invited students to formally apply via a Google Forms survey (Appendix B), with a follow-up email one week before the application deadline. The form required students to provide their name, email, and a commitment to attending all sessions. Applicants also submitted a brief paragraph describing their interest in the course.

Session coordination

We required students to attend all seven CPIM sessions in person. Rooms were equipped with on-demand AV support. Each session began with 45-60 minutes of discussion led by invited speakers, who were encouraged to shape the conversation as they preferred, though we asked them to address the following: What is your role within your organization/what health activism do you participate in? What are the challenges faced by communities that you work with and/or represent? Are there opportunities for students to more directly engage with your work?

Following speaker-led discussions, we devoted the remaining time (30-45 minutes, a total of 90 minutes) to student-only discussions. In the first session of CPIM, we collaborated with students to establish guiding principles for these discussions, including “Call in, don’t call out” (assume best intentions during the discussion, inquire why someone might feel a certain way instead of criticizing their perspective). “One speaker, one mic” (allow only one student to speak at a time). “Avoid reductionism” (encourage students to be specific to the language of the discussion, and avoid generalizations). “Know your place, know your space, know yourself, know your worth” (students should speak based on their experiences and appreciate each other’s unique perspectives and contributions to a discussion).

During the student-only discussion, we invited students to freely share their thoughts on the lecture and topic more broadly. To support participation, we used the following prompts as needed: general thoughts and feelings about the speaker’s lecture, students’ baseline understanding of the topic before the lecture, discussion on how students’ lived experiences relate to the lecture, and experiences or lack of experience in medical school on topics related to the speaker’s lecture.

Specific discussion points were kept confidential. However, we encouraged students to share resources on discussion topics with each other using the course GroupMe.

Community engagement projects

As part of the new iteration of CPIM, we implemented community engagement projects. Over the duration of the course, we asked students to identify a community organization to partner with in some service-oriented capacity. Microgrants were available to support students in need of transportation assistance. We dedicated the final CPIM session of the year to a discussion on students’ community engagement projects over the course.

Session evaluation

Students in the 2023 and 2024 cohorts each completed two surveys: one at the start and one at the end of the course. Both pre- and post-course surveys used five-point Likert scales to assess (a) students’ understanding of that year’s topics, and (b) students’ willingness to integrate health advocacy into their clinical careers. We consulted with the Weill Cornell Office of Medical Student Research in developing effective research questions. This study received IRB exemption from the Weill Cornell Medicine Institutional Review Board.

Likert scale response items are described below. Pre and post-surveys from 2023 asked students to rate how much they agreed with statements on feeling confident in topics covered by invited speakers. Options included (1) Strongly disagree, (2) Disagree, (3) Neither agree nor disagree, (4) Agree, and (5) Strongly agree. Pre and post-surveys in 2023 included 11 questions in total.

For 2024, pre- and post-surveys asked students to rate how confident they felt in their knowledge of topics covered by the course. We set the scale as follows: (1) Not confident at all, (2) Slightly confident, (3) Moderately confident, (4) Very confident, and (5) Completely confident. Course leadership maintained the Likert scale for responses to surveys, while changing the questions from “agreement” on confidence to “confidence” questions to more directly assess perceived competence in a topic. Surveys also provided space for open-ended feedback to improve the course in the future. Pre- and post-surveys in 2024 included 11 questions in total.

We used Wilcoxon rank-sum tests to assess differences in pre- and post-CPIM ratings between groups, with a Bonferroni correction applied to adjust for multiple comparisons. Because surveys were anonymous and not paired, pre- and post-course responses were treated as independent samples. Analyses included all available survey responses at each time point. No imputation was performed for missing data. All quantitative analyses were performed using Stata version 18.5 (StataCorp., College Station, TX, USA).

Qualitative data from open-ended survey responses were analyzed using an inductive thematic approach. Responses were first reviewed in full by members of the course leadership team to familiarize themselves with the data. The team then met to discuss recurring ideas across responses and collaboratively developed a set of themes that reflected students’ shared experiences and perceptions.

## Results

We launched the new iteration of the CPIM course in 2023. In 2023, nine students enrolled in and completed the course. In 2024, 21 students enrolled, with 15 completing the course. All enrolled students were first-year medical students completing their pre-clinical curriculum at Weill Cornell Medical College.

In both years, most speakers were community members with deep activism in their respective topics. Two speakers in 2023 and four speakers in 2024 were healthcare professionals (MDs). Three speakers in 2023 and two speakers in 2024 prepared formal presentations for their discussion, while the remaining speakers provided an informal overview of their topics and opened the floor to dialogue. Students expressed satisfaction with the format of speaker presentations.

Post-course ratings were higher than pre-course ratings across all survey items in 2023 and 2024. In 2023 (Table 1), post-course ratings were significantly higher than pre-course ratings (p < 0.001), from 2.71 to 4.69 on the five-point Likert scale. At the start of the CPIM in 2023, students expressed the least confidence in single-payer healthcare, indigenous health, and healthcare for incarcerated populations. Confidence in these three topics improved significantly at the end of CPIM (p < 0.001), with the greatest improvement in understanding single-payer healthcare. Pre and post-survey results for individual questions in 2023 are shown in Table 1.

**Table 1:** 2023 CPIM pre- and post-survey confidence ratings. Mean and standard deviation (SD) ratings for each pre- and post-course survey item completed by students in the 2023 Community Perspectives in Medicine (CPIM) cohort (n = 9 pre-survey, n = 9 post-survey). Items were phrased as agreement with confidence statements and rated on a five-point Likert agreement scale (1 = strongly disagree to 5 = strongly agree) and reflect self-reported confidence in describing health equity and advocacy topics across course sessions. Pre- and post-survey means were compared using Wilcoxon rank-sum tests, with corresponding Z-statistics and p-values reported; asterisks (*) indicate items meeting the Bonferroni-adjusted significance threshold (p < 0.0022 (0.05/22)).

2023 CPIM data	Mean (SD)		Z-statistic	P-value
	Pre	Post		
N	9 (50.0%)	9 (50.0%)		
I feel confident in describing a single-payer healthcare system*	2.111 (1.054)	4.778 (0.441)	-3.619	<0.001
I feel confident in describing specific ways that a single-payer healthcare system addresses inequities*	2.667 (1.225)	4.889 (0.333)	-3.325	0.001
I feel confident in naming a few specific barriers folks with chronic disabilities face	3.333 (1.225)	4.556 (0.527)	-2.186	0.029
I can name a few ways that I can help my patients with chronic illness feel more autonomous*	2.778 (0.833)	4.444 (0.726)	-3.100	0.002
I feel confident in my knowledge of barriers to healthcare that indigenous groups native to my local community face*	2.111 (0.928)	4.556 (0.527)	-3.662	<0.001
I feel confident in my understanding of the historical context that underlies disparities in the health of indigenous communities*	2.778 (1.093)	4.667 (0.500)	-3.310	0.001
I feel confident in speaking about the impact of mass incarceration and the prison industrial complex on healthcare*	3.111 (1.269)	4.889 (0.333)	-3.230	0.001
I feel confident in my understanding of the health issues affecting the incarcerated population in and around New York City*	2.333 (0.866)	4.667 (0.500)	-3.694	<0.001
I feel confident defining what the police state is and its impact on health institutions*	2.778 (0.972)	4.778 (0.441)	-3.489	<0.001
I can name a few specific ways that academia and healthcare are complicit in upholding the police state*	2.778 (0.833)	4.556 (0.726)	-3.200	0.001
I feel confident in knowing how to work with a community and what it means for healthcare workers to engage in health advocacy*	3.111 (1.054)	4.778 (0.441)	-3.394	0.001

In 2024, the average confidence rating significantly increased (p < 0.001), with scores rising from 2.62 to 3.72 on the five-point scale. At the start of the CPIM in 2024, students expressed having the least confidence in political advocacy in medicine, LGBTQ+ healthcare, and healthcare in humanitarian crises. Confidence in understanding of political advocacy in medicine and LGBTQ+ healthcare increased significantly at the end of the CPIM in 2024 (p < 0.001), with the largest improvement in understanding the current landscape of political advocacy in medicine. Pre and post-survey results for individual questions in 2024 are shown in Table 2.

**Table 2:** 2024 CPIM pre- and post-survey confidence ratings. Mean and standard deviation (SD) ratings for each pre- and post-course survey item completed by students in the 2024 Community Perspectives in Medicine (CPIM) cohort (n = 15 post-survey; 21 pre-survey). Items were phrased as confidence questions and rated on a five-point Likert confidence scale (1 = not at all confident to 5 = very confident) and reflect self-reported confidence in describing health equity and advocacy topics across course sessions. Pre- and post-survey means were compared using Wilcoxon rank-sum tests, with corresponding Z-statistics and p-values reported; asterisks (*) indicate items meeting the Bonferroni-adjusted significance threshold (p < 0.0022 (0.05/22)).

2024 CPIM data	Mean (SD)		Z-statistic	P-value
	Pre	Post		
N	21 (58.3%)	15 (41.7%)		
How confident do you feel in describing what a single-payer healthcare system is?*	2.571 (0.746)	3.533 (0.640)	-3.425	0.001
How confident do you feel in describing specific ways that single-payer healthcare systems can address inequities?*	2.429 (0.676)	3.667 (0.724)	-4.118	<0.001
How confident do you feel describing the ways in which medical infrastructure is affected in humanitarian crises?*	2.238 (1.221)	3.600 (0.910)	-3.163	0.002
How confident do you feel in speaking with fellow classmates and faculty about healthcare in humanitarian crises?	2.381 (1.244)	3.600 (0.910)	-2.871	0.004
How confident do you feel in describing the benefits of community health clinics in underserved communities?*	3.286 (0.902)	3.800 (0.941)	-1.680	0.093
How confident do you feel in describing specific challenges faced by community health clinics, both from a provider and patient standpoint?*	2.571 (0.811)	3.667 (0.900)	-3.268	0.001
How confident do you feel in naming examples of political advocacy in medicine, both past and present?*	2.238 (0.995)	3.733 (0.884)	-3.824	<0.001
How confident do you feel in describing specific ways physicians can get involved in advancing political advocacy in medicine?*	2.238 (0.889)	3.800 (0.775)	-4.160	<0.001
How confident do you feel in describing the specific barriers faced by folks seeking gender-affirming care?*	2.190 (0.750)	3.733 (0.799)	-4.293	<0.001
How confident is your understanding of the social and economic factors that contribute to health disparities?	3.381 (0.865)	4.133 (0.516)	-2.740	0.006
How confident do you feel in your ability to provide culturally sensitive care to diverse patient populations?	3.381 (0.740)	3.667 (0.816)	-1.093	0.274

Community engagement projects for CPIM students varied widely based on student interests and organizational partnerships. Several students reported developing educational materials for local harm reduction programs, assisting student-run community clinics, and collaborating with advocacy groups centered on single-payer healthcare.

Qualitative responses on the impact of CPIM community engagement projects highlighted students’ increased motivation to incorporate community voices into their future healthcare advocacy. Students expressed that having a structured project led to a realization that community-based work is possible within a medical student’s schedule. Students also described establishing longitudinal relationships with community partners, including contacting these organizations to teach additional medical students about specific health justice issues.

“It [community engagement projects] made me realize that doing impactful advocacy work is truly achievable in our role as medical students.”

“Getting involved in a community engagement project through CPIM helped me realize how important it is to consider and involve yourself with communities you intend to advocate for.”

“It [community engagement projects] made me want to continue to incorporate community engagement more into my medical school goals and my career.”

## Discussion

CPIM was originally developed to address critical gaps in medical education concerning SDOH and health advocacy [10]. In redesigning the course, we aimed to deepen first-year medical students’ understanding of health justice issues and enhance their confidence in advocacy through a combination of lectures by community health activists, student-led discussions, and a novel community engagement project. Similar approaches in structural competency and community-engaged curricula have been shown to improve students’ ability to recognize structural influences on health and increase comfort discussing inequity [11,12].

CPIM successfully enhanced students’ confidence in understanding and engaging with a range of health equity topics and advocacy. Quantitative data demonstrated significantly higher post-course confidence ratings compared to pre-course ratings. Qualitative feedback further underscored the program’s impact, with students reporting increased motivation to pursue advocacy and a stronger connection to marginalized communities. The introduction of community engagement projects was particularly impactful, helping students realize the feasibility of incorporating advocacy work into their demanding schedules and fostering longitudinal relationships with community organizations, topics that medical students value [13]. These outcomes mirror prior findings in service-learning and community-based pathways, where structured community engagement strengthened learner motivation and deepened understanding of SDOH [14,15]. Our results also align with evaluations of structural competency and equity-focused curricula, which demonstrate that early, deliberate exposure to structural drivers of health improves students’ confidence in addressing inequity and enhances their readiness to engage in justice-oriented clinical reasoning [16].

Taken together, these findings suggest that CPIM supports the development of a more socially attuned approach to health justice and advocacy among early medical learners by strengthening students’ confidence, readiness, and motivation to engage with equity-focused issues. By providing protected time and a peer-facilitated small-group environment to examine complex, sometimes politicized health issues, CPIM reinforces the value of structured spaces for critical dialogue, an approach similarly shown to improve learners’ comfort discussing racism, bias, and structural inequity in other curricular models [17]. The combination of community partnership, student leadership, and reflective discussion appears to be a particularly powerful mechanism supporting these outcomes.

The development and implementation of CPIM, particularly its restructured format, offer several additional valuable lessons. First, the power of community voices: involving activists and CBO leaders offers students real-world insight into health disparities, reinforcing the value of community-engaged medical education. This aligns with research emphasizing the value of community-engaged medical education and partnerships with community organizations to improve students’ understanding of SDOH [1,18]. Second, structured engagement deepens learning: the community project shifted students from passive observers to active participants, echoing the benefits of experiential learning in health equity [19]. Lastly, logistical considerations: effective planning, including speaker honoraria and student meals, supports smooth execution and encourages participation.

Limitations

As a student-run, non-credit elective at a single institution, the findings on CPIM presented here may not be generalizable to broader medical education contexts, particularly given the self-selected nature of participants and reliance on local community organizations. The evaluation relied on self-reported confidence levels, which, while useful, do not objectively measure knowledge gains or long-term behavioral changes. Furthermore, the change in survey wording across 2023 and 2024 limits comparability between the two years, though both survey tools measure students’ self-perceived confidence. The absence of a control group (i.e., students who did not participate in the course) further limits causal inferences. Finally, attrition in the 2024 post-course survey may have introduced bias in pre-post comparisons, as analyses were limited to students who completed the course.

Future directions

Future research should focus on the long-term impact of such electives, tracking students’ engagement in advocacy and community health initiatives throughout their medical careers. This would provide stronger evidence of the lasting benefits of these programs. Additionally, the development and validation of standardized evaluation tools to assess competency in health equity and advocacy would benefit the field by allowing for more comparable evaluations across different programs and institutions. There is ongoing work to guide programs in determining which outcomes best reflect areas of programmatic need and impact in equity-focused curricula.

## Conclusions

Our findings build on the growing literature that emphasizes experiential and service-learning models as effective approaches to teaching SDOH. By grounding course content in the CDC’s SDOH framework and linking didactic sessions to community partnerships, CPIM aligns with calls for more structured, community-engaged methods of health equity education. CPIM’s elective model holds significant potential for future development and broader implications for medical education and curricula. The success of CPIM advocates for the integration of similar community-centered, health justice-focused educational components into core medical school curricula nationwide, rather than solely as elective opportunities as they are typically offered. This aligns with calls to ensure future physicians are competent in addressing SDOH and health inequities, potentially through required experiential learning practicums responsive to community needs. To support wider implementation, medical schools should invest in faculty development to equip educators with the skills and knowledge to teach SDOH and health advocacy effectively.

## Appendices

Appendix A. Email template to speakers

**Table 3:** Sample speaker recruitment email.

Component	Content
Greeting	Hello,
Introduction	I hope this email finds you doing well. My name is [X], and I am a medical student at [X]
Course description	I am coordinating an elective at Weill Cornell called “Community Perspectives in Medicine,” where we aim to engage students on health equity-related topics that are not a significant part of the medical curriculum. One of the topics we hope to cover is [X], with a focus on groups pursuing this work at a community level in New York City
Invitation to speak	I wanted to see if you would be available to speak to students in our elective on this topic. If you are available, the session would be on [insert date and time]. [Include available time slots]
Logistics	We would set up a Zoom call for you to join at that time, or you are welcome to join in person. We can also provide compensation in recognition of your time, likely in the form of a gift card
Closing statement	I believe our medical students would greatly benefit from the insights, experiences, and perspectives you have to share. Looking forward to hearing from you. Many thanks in advance for considering this!

Appendix B. CPIM application

**Table 4:** Sample Community Perspectives in Medicine (CPIM) application.

Community Perspectives in Medicine (CPIM) Elective Application (2024)	
Description	The Community Perspectives in Medicine (CPIM) elective provides an opportunity for first-year medical students to learn about providing humanistic care from the perspective of community advocates and activists. The elective focuses on encouraging discussions related to health inequities while building partnerships with community-based organizations. CPIM consists of a series of 1.5-hour sessions; the first hour of each session is a conversation with a community-based organization focused on the challenges they face in healthcare settings, and the last half-hour is a student-only debrief discussion. Dinner is provided at each session. This year’s sessions include: 09/24 – Introductory Session 10/01 – Single Payer Healthcare/Medicare for All 11/08 – Chronic Illness 11/15 – Reproductive Justice 11/22 – Detainee Health 11/29 – Health in Humanitarian Crises 11/05 – Community Surveillance + Goodbyes. The order of sessions might shift depending on speaker availability. Sessions will run on Tuesdays from 6–7:30 PM. Applications are due Tuesday, Sept 10th
Name*	
Which pronouns do you use? (optional)	
School email*	
Can you commit to all of the above sessions?*	Yes/No/I may need to miss 1–2 sessions and will elaborate in the Questions/Comments section below
Please tell us why you believe you would be a good addition to the CPIM elective (max 150 words)*	
Any dietary restrictions/allergies? (Check all that apply)	Vegetarian/Vegan/Kosher/Halal/Gluten-free/None
Typically, CPIM is held in-person, on campus. Please let us know if you have any accessibility requests:	
Questions/Comments	
